# Neuropsychiatric Manifestations in Systemic Lupus Erythematosus: Psicosis as a Clinical Challenge in a Case Report and Updated Literature Review

**DOI:** 10.1192/j.eurpsy.2025.1674

**Published:** 2025-08-26

**Authors:** E. Abalde García, E. Lanchares Balboa, K. Muñoz Rodriguez, L. C. Ortega Sandoval

**Affiliations:** 1 Hospital Álvaro Cunqueiro , Vigo, Spain; 2 Psychiatry, Hospital Álvaro Cunqueiro, Vigo, Spain

## Abstract

**Introduction:**

Systemic lupus erythematosus (SLE) is a multisystem autoimmune disease that affects the central nervous system (NP-SLE) in approximately 30-40% of patients, with psychosis being one of the less common yet very serious manifestations, occurring in about 2-3.5% of cases. Diagnosing psychosis in SLE can be challenging, requiring careful integration of medical history with appropriate complementary tests (immunological, CSF, neuroimaging…) while considering other possible differential diagnoses.

**Objectives:**

To highlight the importance of considering this condition (as well as other rare causes of neuropsychiatric involvement) in patients presenting with atypical psychotic symptoms accompanied by cognitive and behavioral disturbances.

**Methods:**

Presentation of a clinical case and literature review.

**Results:**

A 69-year-old female presents with a complex medical history, including melanoma, meningioma, and cerebral microangiopathy. Since 2019, prolonged activated partial thromboplastin time (aPTT) and lupus anticoagulant have been observed. In 2020, she developed neurological symptoms such as blurred vision, diplopia, dizziness, and instability. In December 2022, she consults for depressive symptoms and memory problems. June 2023, she was admitted to Psychiatry ward for a psychotic episode characterized by poorly structured and apparently fluctuating delusional symptoms. There was also evident disorganization, confusion, incoherent behavior, memory deficits, and functional decline. During the hospitalization, her condition initially worsened, with bizarre behaviors fluctuating, requiring mechanical and pharmacological restraint on several occasions. Blood tests showed bicitopenia (leukopenia and thrombocytopenia), along with renal insufficiency. Antiphospholipid syndrome was confirmed. Subsequently, her delusional symptoms remitted, and she was discharged. A second scheduled admission in internal medicine followed for diagnostic clarification during which SLE (ANA+, SSA, SSB, anti U1RNP, anti Sm D, and anti-DNA+) and antiphospholipid syndrome were confirmed. Hydroxychloroquine and anticoagulation (Sintrom) were initiated.

She currently remains under follow-up in both Internal Medicine and Psychiatry with functional, cognitive, and emotional improvement, allowing for the reduction of antipsychotic and antidepressant medication without relapse. A cognitive decline has been ruled out by the dementia unit.

**Conclusions:**

In the case of SLE and its neuropsychiatric manifestations (NP-SLE), there are no specific markers, making detailed medical history, thorough screening, and differential diagnosis essential. Appropriate use of complementary tests and close coordination with other medical specialties are crucial, requiring a multidisciplinary and holistic approach.

**Disclosure of Interest:**

None Declared

